# The Role of Osteopathic Manipulative Treatment in Osteoarthritis: A Scoping Review

**DOI:** 10.7759/cureus.74440

**Published:** 2024-11-25

**Authors:** Veenah Stoll, Jennifer Trube, Timothy Johnson, Jake Darbhanga, Rohit S Mehra

**Affiliations:** 1 Osteopathic Medicine, Nova Southeastern University Dr. Kiran C. Patel College of Osteopathic Medicine, Clearwater, USA; 2 Internal Medicine, Lakeland Regional Health, Lakeland, USA; 3 Emergency Medicine, Hospital Corporation of America Florida Brandon Hospital, Tampa, USA

**Keywords:** alternative medical therapies, complimentary medicine, geriatric, nonsteroidal anti-inflammatory drugs (nsaids), osteoarthritis (oa), osteopathic manipulative medicine (omm), osteopathic manipulative treatment (omt), osteopathic principles and practice (opp), physical medicine and rehabilitation, total joint arthroplasty

## Abstract

Osteoarthritis (OA) is one of the most common joint disorders in the United States. As no whole body, curative treatment exists, conservative, often multimodal, treatment goals are used first with aims to decrease pain and improve function in daily life. This scoping review seeks to understand how incorporating osteopathic manipulative treatment (OMT) in the management of OA can affect patient outcomes, specifically pain and mobility. This was explored by searching CINAHL, EBSCO, Web of Science, EMBASE, and Ovid MEDLINE(R) databases on 11/18/2022. Search terms included: [(“osteoarthr*") OR ("degenerative arthr*") OR ("degenerative joint disease") OR ("noninflammatory arthr*") OR ("osteo arthr*")] AND [(”osteopath*") OR (omt) OR (omm) OR (“manipulat* medicine”)]. Inclusion criteria were articles that identified the use of OMT or manipulation in the OA treatment of adults. A total of 488 articles were extracted. After duplicates were removed, 353 articles remained. Seven articles were included; five primary articles and two review articles. Across five eligible primary articles, 186 patients were identified. Articles were assessed for the use and effectiveness of an OMT treatment plan for pain reduction and improved outcomes, including but not limited to, range of motion (ROM), functionality, and quality of life. OMT is a safe and effective method able to be used alone or in combination with other treatments for OA. There is reasonable preliminary evidence to suggest OMT can be used to provide statistically significant improvement in pain, joint stiffness, ROM, functionality scores, and physical exam findings, but future large-scale clinical trials are recommended.

## Introduction and background

Osteoarthritis (OA) is the most common joint disorder in the United States with an estimated prevalence of 32.5 million adults per year. OA is most commonly diagnosed in adults 55-64 years of age, but it is a disease that can affect a person at any age [1]. OA is a multifactorial degenerative process that destructs non-cartilaginous structures like subchondral bone, synovium, and periarticular cartilage often leading to chronic pain and impaired daily functioning [2]. Despite being a common, debilitating disease without any systemic curative treatments, total joint arthroplasty (TJA) remains the only definitive treatment for specific joints affected by severe OA. Surgical treatment with TJA may eliminate bone spurs and damaged cartilage but can still leave patients with chronic pain and functional impairment. First-line pharmacological treatment of OA includes non-steroidal anti-inflammatory drugs (NSAIDs), used both acutely and chronically to reduce symptoms. However, there are several side effects associated with chronic oral NSAID use [2]. These side effects can include gastritis, peptic ulcers, chronic kidney disease, acute kidney injury, heart failure, meningitis, and cognitive dysfunction, all of which lead to a significant number of hospitalizations and fatalities each year [3].

Non-pharmacologic treatments are a favorable alternative and include modalities, such as physical therapy (PT), exercise, and osteopathic manipulative treatment (OMT). When used effectively, OMT decreases stiffness and pain and increases blood and lymphatic flow [4-6]. However, despite its use worldwide, the literature lacks high-quality clinical trials demonstrating OMT effectiveness in OA treatment. This paper approaches the use and OMT effectiveness in OA treatment in adults using the framework for a scoping review, as outlined by the Preferred Reporting Items for Systematic Reviews and Meta-analysis Protocols Extension for Scoping Reviews (PRISMA-ScR) guidelines [7]. The following question was addressed: What research has been published that uses OMT as an intervention for OA treatment in adults? We hypothesize that OMT will minimize pain and improve functional range of motion (ROM) through increased stretching, muscle strength, and the alleviation of restrictions in the treatment of OA. We believe a scoping review is best suited to critically analyze the status of the literature in these areas with recommendations for future research.

## Review

Materials and methods

Stage 1: Identifying the Research Question

The research question that directed this scoping review was: What research has been published that uses OMT as an intervention for treating OA?

Stage 2: Identifying Relevant Studies

Our protocol was drafted using the PRISMA-ScR guidelines [7]. CINAHL, EBSCO, Web of Science, EMBASE, and Ovid MEDLINE(R) databases were searched on 11/18/2022 to identify relevant data. The search strategies were reviewed by an experienced medical librarian and further refined through a discussion of criteria. The final search strategy included terms [(“osteoarthr*") OR ("degenerative arthr*") OR ("degenerative joint disease") OR ("noninflammatory arthr*") OR ("osteo arthr*")] AND [(”osteopath*") OR (omt) OR (omm) OR (“manipulat* medicine”)]. No year restrictions or dissemination age restrictions were placed. Only journal articles with full-text availability published in English were included for ease of access and limited translation services were available to the researchers.

Stage 3: Study Selection 

Studies yielded from the search were imported into Rayyan software where duplicate articles were removed. Study criteria included adults over 18 diagnosed with OA, and study objectives assessing OMT use in OA treatment. Table 1 shows the population, concept, and context (PCC) eligibility criteria. Articles that described patients with diagnoses of active cancer, acute or traumatic injury, rheumatoid arthritis, inflammatory joint disease, hypertrophic osteoarthropathy, low back or neck pain as a primary diagnosis, post-infectious sequelae, pregnant patients, patients with Coronavirus Disease 2019 (COVID-19) or active infections, and patients with a history of joint replacement for the joint studied were excluded. Articles were also excluded if manipulation was performed by a physical therapist without osteopathic manipulative medicine training or if the only treatment was chiropractic treatment.

**Table 1:** PCC eligibility criteria PCC: Population, concept, context; OA: Osteoarthritis; COVID-19: Coronavirus disease 2019; OMT: Osteopathic manipulative treatment

PCC	Inclusion Criteria	Exclusion Criteria
Population	Human adults over the age of 18 years with the diagnosis of OA	Patients with active cancer, acute strain, traumatic injury, rheumatoid arthritis, or psoriatic arthritis. Inflammatory joint disease or low back pain as a primary diagnosis. Chronic low back pain due to bulging or herniated disks. Neck pain as a primary diagnosis. Patients currently pregnant. Hypertrophic and other kinds of osteoarthropathy. Patients diagnosed with COVID-19 infection or other active infection. Past or current treatment of chronic joint pain due to rheumatologic disease. History of genetically inherited diseases.
Concept	Articles that describe OMT or manipulation as a treatment for OA. OMT or manipulation performed by an osteopathic or osteopathic physician. Only articles written in English to ensure accurate interpretation. Articles published and available in full-text.	Patients currently treated with injectable/intramuscular medications. History of post-infectious sequelae. History of joint replacement of the joint studied.
Context	Regions with access to osteopathic physicians or osteopaths	Regions who do not have access to holistic medicine. History of chiropractic treatment.

Three reviewers independently evaluated all titles and abstracts to identify relevant studies. Disagreements were reviewed by discussion, and one of the remaining authors was the tie-breaker. Full-text articles were retrieved in portable document format (PDF) or electronic formats. Only articles available for free through the university subscription or the publisher were accepted, but no articles were identified that required further paid access.

Stage 4: Charting the Data

After applying exclusion criteria, the final studies were exported to a spreadsheet. Full-text articles were reviewed by all authors. Extraction fields included authors, samples, purpose, methods, results, and limitations. Data extraction was completed by four authors independently, and the data was finalized and confirmed for consistency by two authors.

Stage 5: Collating, Summarizing, and Reporting

After authors, sample, purpose, methods, results, and limitations were extracted, studies were grouped by research type in table format and narrative format to discuss broad findings. Initial categorization was performed by two authors and quality was checked by the remaining authors. Articles were assessed for the use of OMT in adults and the effectiveness of treatment in pain reduction and improved patient outcomes. The abstraction of data was completed by two authors, independently.

Results

**Figure 1:**
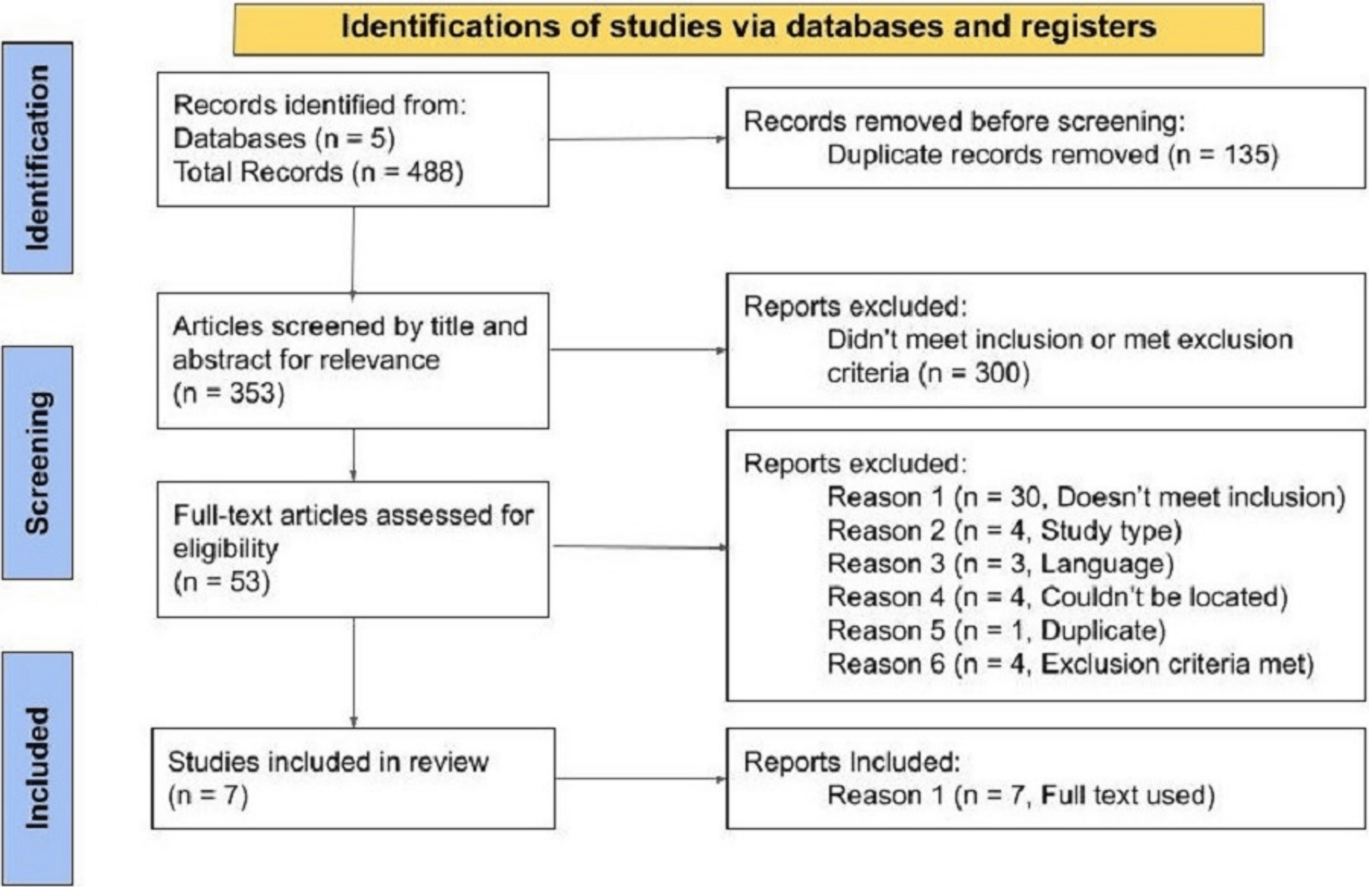
Flow chart of PRISMA selection process used (and numbered) to identify total articles, screening for eligibility and those included PRISMA: Preferred Reporting Items for Systematic Reviews and Meta-Analyses; n: Number

A total of 488 articles were extracted into Rayyan for initial review. After duplicates were removed using Rayyan software and manual review, 353 articles were captured. These articles were reviewed for title and abstract eligibility. After abstracts were reviewed, 53 articles were assessed for full-text eligibility. Seven articles were included in the review (Figure 1). Six additional articles that did not meet the full study criteria were utilized for background information.

The results of this scoping review are summarized in Table 2. This search string identified four clinical trials and one case report that provided primary data [6,8-11]. The other two articles were review articles that outlined an osteopathic approach to assessing and evaluating knee pain [5,12]. Across five studies, 186 patients received OMT for OA and were analyzed for improvements in ROM, pain scores, and physical function testing.

**Table 2:** Synthesis of articles detailing the purpose, samples, methods, results, and limitations for articles OA: Osteoarthritis; MFR: Myofascial release; ROM: Range of motion; MET: Muscle energy technique; FPR: Facilitated positional release; VAS: Visual analogue scale; OMT: Osteopathic manipulative treatment; CKP: Chronic knee pain; CSP: Chronic shoulder pain; CNP: Chronic neck pain; CLBP: Chronic lower back pain; WOMAC: Western Ontario and McMaster Universities Osteoarthritis Index; NSAID: Nonsteroidal anti-inflammatory drug; IT: Iliotibial; LAS: Ligamentous articular strain

Reference	Purpose	Sample	Methods	Results	Limitations
Van Manen et al., 2012 [5]	A review article to analyze nonoperative management for knee OA and surgical procedures less invasive than total knee arthroplasty	No methods were listed for review.	No clinical evidence was provided.	Normalizing myofascial and lymphatic restrictions through techniques, such as counterstain positioning, lymphatic pump, trigger point injections, soft-tissue massage, and MFR may assist in managing knee OA.	There was no information regarding the method of literature review.
Jardine et al., 2012 [6]	A clinical trial to determine if fascial releases along the arterial pathway and balancing of diaphragmatic tensions can influence the vascular supply, dynamic balance, knee ROM, and symptoms in patients with radiographic knee OA	A convenience sample of 30 participants between the ages of 48 and 80 years with symptomatic and radiographic knee OA were recruited through local healthcare professionals.	A final-year osteopathic student with 20 years of experience as a physiotherapist conducted osteopathic evaluations and treatments, using ultrasound/Doppler recordings of the resistive index of the superficial femoral artery, active knee flexion ROM, step test for balance, and the VAS symptom rating. Techniques included normalizing the thoracic diaphragm, tentorium cerebelli, pelvic floor, iliac fascia, and global femoral artery, as well as balancing the three diaphragms.	Significant pre/post-test main effects were found for ROM, balance, and symptom measures. Greater knee flexion ROM, greater number of steps taken during the step test, and self-reported symptoms were reduced during the post-test evaluation compared to pretest evaluations.	Small patient population, possible Hawthorne effect or effect of therapeutic touch was not addressed with a control group.
Addala et al., 2013 [8]	A clinical trial to examine the effect of MET on pain and ROM in a population with OA of the knee	30 OA subjects between the ages of 45-60 years; 15 subjects in the control group, 15 in the muscle energy group	Unspecified MET was used on 15 patients and compared to conventional therapy. Outcome measures were ROM and VAS.	The experimental group showed significant improvement compared to the control group. MET played an important role in decreasing pain and increasing ROM in OA in this study population (p=.001).	Small sample size, treatment length, and unspecified MET reduce replicability.
Altınbilek et al., 2018 [9]	A clinical trial to compare the efficacy of OMT with exercise versus exercise treatment alone in knee OA	85 patients were recruited aged 40-70 years. 44 patients were assigned to an OMT and exercise group, and 41 patients were assigned to an exercise group alone.	Exercise program included quadriceps isometric strengthening, straight leg lifting, iliotibial band, hamstring stretching, strengthening abductor and adductor muscle of the hip, and stretching exercises. This was applied as 3 sets of 10 repetitions, two days a week for a total of four clinic visits. Patients were recommended to perform exercises two times a day at home. OMT group received 3-minute mobilization and 3-minute compression for bilateral patellofemoral and tibiofemoral joint respectively with 1-minute intervals in addition to the exercise program.	Functional improvement (p<0.05) and pain relief (p<0.05) were significantly higher in the exercise and OMT group.	Limited population and unclear methods of OMT present limited replicability.
Datta et al., 2017 [10]	A review article that presents the epidemiology, clinically relevant anatomy, physiology, and major risk factors associated with common knee pain conditions	No patient data was recorded.	The article proposes an osteopathic approach to knee pain treatment using various OMT modalities.	MET or FPR can be used for the tibiofemoral joint. Counterstrain to the medial and lateral knee. High velocity low amplitude to the fibular head.	This is an approach described without patient data to corroborate effectiveness.
Rotter et al., 2022 [11]	A prospective cohort study to observe changes after OMT in addition to routine care regarding pain, functionality, and quality of life in patients with four chronic musculoskeletal pain diseases	A prospective cohort study of 40 patients with 10 patients in each of the four groups: CKP, CSP, CNP, and CLBP	Patients suffering from CKP, CSP, CNP, and CLBP received six OMT sessions in addition to routine care. Musculoskeletal, visceral, and craniosacral systems were treated in a standing, sitting, or lying position, with or without active participation by a medical doctor specializing in orthopedic surgery and who completed a 5-year part-time OMT training program with an MSc degree in osteopathy and long-term experience in OMT. Outcomes were assessed at 12, 26, and 52 weeks.	After 26 weeks of OMT sessions improvement was seen in the VAS pain score and total WOMAC scores in the whole population. No adverse effects were observed, and the effects of treatment persisted with similar pain reduction at 52 weeks.	Small number of patients per diagnosis of OA. No control group included. 60% of patients had previously received OMT in the past, with 30% in the knee pain subgroup having received OMT for this complaint greater than one year ago.
Zaidi et al., 2006 [12]	A case report to determine the effects of OMT and low dose NSAID and orthotics on reducing pain and increasing the ROM in a 23-year-old female patient with OA of the left foot	The study involved a case report of a 23-year-old female with OA of the left foot.	Weekly 30-minute LAS treatments for three weeks were administered to the feet, ankles, legs, thighs, and knees bilaterally, and the sacrum, with detailed attention to the ligaments of the left ankle and knee IT bands on both legs and the interosseous membrane between the tibia and fibula.	The patient reported a significant decrease in pain after the first few treatments, despite a reported increase in physical activity. Pain in her right foot went from 2/10 to complete relief, while pain in the left foot went from 4/10 to 1/10. Knee pain was still present but no longer radiated. She has increased ROM in her left foot so that it is equal to the right foot.	Population was limited to one patient. Only one modality was used.

One of the clinical trials by Altinbilek et al. compared exercise alone versus exercise with OMT mobilization and compression techniques for the bilateral patellofemoral and tibiofemoral joints. There were statistically significant improvements in physical exam findings including warmth, effusion, crepitus, patellar grind test, valgus and varus stress tests, Apley compression test, Apley distraction test, knee circumference, and knee ROM (p<0.05) [9]. There were also statistically significant clinical improvements in Western Ontario McMaster Questionnaire (WOMAC) pain scores, joint stiffness, physical function scores, Visual Analogue Scale (VAS), and 50m walking time scores in the patients between the two groups (p<0.05) [9].

Addala et al. explored the effects of three weeks of muscle energy technique (MET) on pain and ROM in patients with knee pain secondary to OA [8]. There were 30 patients studied, with half receiving conventional therapy and the other half receiving MET for six weeks. Upon completion of treatment, there was a statistically significant decrease in knee pain (p=0.01) with increased ROM (p=0.01) in study group patients relative to the control group [8]. Additionally, the experimental group had more significant improvement in all parameters than the control group.

An observational trial by Rotter et al. studied individually tailored 45-minute OMT sessions for chronic knee pain in ten patients [11]. The musculoskeletal, visceral, and craniosacral systems were treated at 3-4 week intervals in addition to routine care. Pain, function, and quality of life were assessed with a VAS from 0-100mm and a standardized questionnaire. After 26 weeks, significant VAS score clinical benefit of >26.5mm and total WOMAC score reduction were achieved with a 95% confidence interval [11]. When outcomes were reassessed at 52 weeks, patients reported persistent total WOMAC score improvements slightly greater than those found at 12 and 26 weeks.

Jardine et al. demonstrated statistically significant improvement in ROM, balance, and symptoms reported by the patients in the treatment group (p<0.05) [6]. This was achieved through normalizing the diaphragms, tentorium cerebelli, pelvic floor, iliac fascia, and global femoral artery release. An improvement was also noted in the restrictive index of the superficial femoral artery measured by color and pulse wave Doppler ultrasound of patients in the treatment group (p<0.008) [6]. A limitation noted was a concurrent statistically significant improvement in ROM, balance, and reported symptoms in the control group who only received a thorough osteopathic assessment (p<0.05) [6].

Ligamentous articular strain (LAS) performed on the feet, ankles, knees, and sacrum was shown to improve pain and ROM in a case report of left foot pain secondary to OA [12]. The improvement persisted despite an increase in the patient's physical activity. The patient reported their foot pain decreased from a 4/10 to a 1/10, and the ROM of the affected foot increased to match that of the unaffected foot [12].

Two articles were included that exhibited secondary data, analyzing non-pharmacological approaches to knee pain. Datta et al. proposed an osteopathic approach to knee pain. They discussed the use of various OMT modalities including MET, facilitated positional release (FPR), counterstrain, and high-velocity low amplitude (HVLA) techniques to treat OA of the knee. Van Manen et al. discussed using OMT including myofascial and lymphatic normalization through counterstrain, lymphatic pumps, soft-tissue, and myofascial release (MFR) to assist in managing knee OA [5].

Discussion

Based on preliminary studies, OMT is a promising option for managing patients with OA. OMT can be used alone or as part of a multimodal treatment plan to improve patient outcomes. Of the five articles with primary data points, chronic pain was reduced in all articles, and four of five articles reported improvements in pain and ROM.

OMT addresses the dysfunction of surrounding muscles, ligaments, and fascia associated with OA. Realignment of the joint and reduction of capsular adhesions are the proposed mechanisms for reducing pain and improvement in ROM. The common osteopathic techniques include MET, counterstrain, soft tissue massage, LAS, and MFR, which have been shown to improve ROM and reduce disease burden [6,8,9,11,12]. These techniques promote the relaxation of the surrounding tissues through forces applied to hypertonic muscles. It was demonstrated that MET, MFR, LAS, soft tissue, and lymphatic pumps, in particular, could provide patients with pain reduction and improvement of symptoms [6,8,12]. It was also suggested that FPR, counterstrain, and HVLA could be used to treat the symptoms of OA through similar justification. However, these modalities have not yet been tested clinically [9,11].

A foundational concept of OMT, “the rule of the artery is supreme,” states, that only when the circulatory and lymphatic systems are optimized, can tissues perform their designated functions most efficiently [6]. Another key principle of OMT is that the body functions as a unit with the inherent capacity to heal and repair itself. Blood flow plays an important role in healing, nutrient delivery, and waste elimination. Therefore, it would be logical if the proposed mechanisms of several OMT techniques could improve function and daily living activities in patients with OA. Lymphatic pumps, normalization of the three body diaphragms, normalization of the tentorium cerebelli, iliac fascia release, and global femoral artery release are thought to improve fascial restriction and balance tension on the blood and lymphatic vessels. When used in combination, these techniques were shown to decrease the restrictive index of the superficial femoral artery measurements on color and pulse wave Doppler [5,6].

Dysregulation of the autonomic nervous system is identified as another dysfunction in OA that has been shown to affect a patient’s perception of pain [5]. Increased sympathetic activity can heighten a patient's sensitivity to pain, reducing the use of the affected joint, and further restricting function over time. OMT is thought to treat these dysfunctions in the nervous system by relaxing the sympathetic through techniques such as rib raising or soft tissue release of the thoracic spine [5]. There is also physiologic evidence to support the chronic pain reduction demonstrated with OMT. A prospective, blinded assessment was performed that collected blood samples from subjects with and without chronic lower back pain over several days. In those same patients, OMT was administered 1-hour before blood collection on day four. Several circulatory pain biomarkers were analyzed including β-endorphin, serotonin, 5-hydroxyindoleacetic acid, anandamide, and N-palmitoylethanolamide. These levels provided quantitative trends while controls accounted for confounding variables. Results demonstrated the greatest changes in β-endorphin, anandamide, and N-palmitoylethanolamide both 30 minutes and 24 hours after OMT were greater in patients with chronic lower back pain versus those without [13].

Through its numerous mechanisms and diverse applications, OMT has demonstrated effectiveness in quelling various causes of chronic pain. One systematic review reported that OMT was significantly associated with reduced pain and disability and improved quality of life compared to standard care [14]. Moderate quality evidence also demonstrated OMT plus exercise was significantly associated with a reduction in pain and disability severity compared to exercise alone [9,14,15]. Similar positive outcomes have been tested in various body regions, including the neck and knees. One randomized controlled trial with a cross-over design compared immediate OMT in chronic neck pain vs delayed OMT in patients with equivalent baseline pain. This study found a significant reduction in average pain and disability. There were also improved secondary outcomes related to sleep, fatigue, and depression without any adverse reactions reported. [16]. Other studies have proposed that a combination of pharmacologic treatment and OMT may be an effective option in the reduction of significant chronic pain caused by OA [17]. These diverse applications of OMT on chronic pain postulate a very similar therapeutic benefit when applied to patients with OA.

OMT is also a promising alternative treatment for OA that can reduce chronic NSAID use and even polypharmacy issues faced by the geriatric population. Gentle modalities, such as MFR or counterstrain, are especially useful in the geriatric population that may be unable to tolerate forceful maneuvers or follow commands required for MET. Other potential populations that could benefit from reduced NSAID use are those with chronic kidney disease, allergies, medication interactions, or other contraindications to NSAIDs. By minimizing pain and improving daily function through the incorporation of OMT, patients may be able to achieve the same desired relief from symptoms with fewer chronic medications. However, further large-scale randomized controlled trials are needed to augment the current literature that shows promising preliminary findings.

There are currently a limited number of high-quality studies published assessing the effectiveness of OMT as a treatment for OA. Based on this gap in the literature, strong clinical recommendations cannot be made at this time. Further research could also be conducted to compare the effectiveness of different OMT modalities for treating OA. Ultimately, OMT is a safe method that can be used alone or in combination with conservative or pharmacologic therapies with the potential to minimize suffering and optimize the treatment outcomes in OA.

This scoping review has several limitations that should be considered when interpreting these results. Simply providing a thorough osteopathic examination can result in a statistically significant phenomenon called therapeutic touch which is well-recognized in the literature [6,18]. As the study done by Jardine et al. demonstrated, therapeutic touch used as a control group during the initial assessment can lead to a statistically significant improvement in ROM and patient outcomes [6]. Therapeutic touch is so significant, that it can sometimes even decrease pain without physical contact [18]. Additionally, only full-text, English articles were included for translational purposes. Cautious interpretation of the positive yet statistically significant data is warranted due to small sample sizes and some studies lack of control groups. These studies without control groups are unable to account for the known benefits of therapeutic touch.

## Conclusions

When managing chronic diseases such as OA, the primary goal should focus on increasing the patient’s quality of life. OMT is a conservative treatment option for OA that can be tailored to an individual based on symptom location, type, and severity to improve pain and function. This research has demonstrated numerous approaches to using OMT in alleviating the undesired symptoms of OA, including, but not limited to MET, MFR, LAS, and lymphatic techniques. OMT showed statistically significant improvement in pain score, joint stiffness, ROM, balance, physical exam findings, and physical function scores in several studies. Future studies could strengthen OMT and OA research by performing various OMT techniques on differing severities and joints with OA on a larger patient population to quantify its impact better.
